# The Nutritive Value of "Standard" Bread
**The Technology of Bread-making.* By William Jago, F.I.C., F.C.S., and William C. Jago, food-manufacturing chemists. London: Simpkin Marshall, Hamilton, Kent and Co., Ltd. 1911.


**Published:** 1911-09-23

**Authors:** 


### DIETETICS.

### THE NUTRITIVE VALUE OF "STANDARD" BREAD.*

### HOW BROWN BREAD DIFFERS FROM WHITE.

Lately there has been a general stimulation of public interest in the subject of bread, its preparation, its nutritive value, and its varieties. We have been treated to an elaborate apology for the use of bread made with "standard flours," and we have been solemnly assured, not indeed by dieticians, but by some members of the medical profession who appended their names to a much-discussed "manifesto" that it is imperatively necessary for the community to eat "standard" bread. We have had a report on bleached flour; we have had statements dealing with the conditions under which baking is carried on in our large cities; and last, but not least, physiologists asserting that white rats fed on offal flours thrive much better than other specimens of the species which have been put on a diet of white bread and milk. It is hardly necessary to say that the public has not been in a position to make up its mind upon the subject. Where doctors disagree to such an extent, and where the subject of disagreement involves such technicalities as those used in bread-making, it is hardly to be wondered that the average layman feels diffident, to say the least, in deciding between the relative claims of white and brown bread.

The nutritive value and digestibility of bread are subjects which have exercised the minds of dieticians from the earliest period. Hippocrates imagined that unfermented bread was necessarily bad bread; Galen supported the view of Herophilus that bread which contained husks of wheat gave rise to colic; Cornaro laid stress on the fact that he ate only "fine bread"; Cardinal du Prat, famous in gastronomic history for his attachment to the dietetic rules of the school of Salern, suggested that the world would be all the better if it fell back on the hundred and one varieties of cheesecakes enumerated by Athenæus in "The Deipnosophists." But it is only since the times of Liebig, when we learned to estimate the true value of an article of diet by judging in how far it fulfils the conditions required of an ideal diet, that we are in a position to differentiate between bad and good bread. Tunnicliffe and Brunton in 1899, experimenting with samples of the best white bread and a wholemeal bread, showed, as conclusively as it was possible to show, that the white bread is more easily digestible than the brown, and that its nutritive value is a fraction higher. It contains relatively more proteid, and of this total proteid content that of white bread is more easily assimilated than that of brown. The Jagos, working with various samples of bakers' bread, arrive at pretty much the same conclusions, while the results of Atwater, Woods, and Snyder, who may claim to be especially the authorities on the dietetic value of bread, support these findings. Taking all these data, the authors of the latest and most exhaustive work on bread-making, which is the subject of this review, frame the following conclusions, which seem

* *The Technology of Bread-making.* By William Jago, F.I.C., F.C.S., and William C. Jago, food-manufacturing chemists. London: Simpkin Marshall, Hamilton, Kent and Co., Ltd. 1911.

eminently reasonable and fair in the circumstances: "White bread is more nutritious than wholemeal or ordinary brown breads. The average best white bread is more nutritious than the second quality, or that made from the darker or low-grade flours. The addition of finely divided bran to white flours lowers the nutritive value of the mixture. The addition of germ in excess to that normally present in wheat increases the nutritive value of the bread."

We now come to the so-called "Standard Bread." At the outset it may be premised, as the authors point out, that the fixing of a standard for the nutritive value of bread is not such a simple matter as the signatories of the notorious manifesto appeared to think. The suggested standard is "at least 80 per cent. of the whole wheat," and the authors instance how irrational such a standard would be in actual practice. The manifesto dogmatically states that the white bread commonly sold in this country is of inferior nourishing quality, but the results obtained by the authors in attempting to test this dictum certainly do not support the contention. It appears that while ordinary white bread gives a percentage of almost six and a half of digested proteid, "standard," on the contrary, gives only a percentage of approximately six. "It follows," they conclude, "that as a source of available proteid 70 per cent. straight run flour makes a more nutritious bread than 80 per cent. standard." Even more remarkable and more convincing are the actual results obtained by putting children on a diet of "standard" bread for a fortnight. It was suggested that such children would immensely prefer the "standard" bread and would thrive better on it; but here again the authors find that the children objected to "standard" bread, ate less of it, and begged to be allowed to return to white bread, and on the change being made there was a general improvement in their appetite and spirits. These results agree closely with those obtained by other experimenters who have given the new fancy a fair trial in the diet of poor children. Snyder, to whom the authors sent the manifesto, expresses himself very strongly, and his letter, which is too long to quote in full, deserves to be read by everyone who is still under the impression that "standard" bread is the most ideal bread for human consumption. "I am satisfied," he writes, "that it is not feasible, from a milling point of view, to include in the flour the offal, or any part, no matter how finely ground; that it results in a poorer quality of bread physically, and lowers the total digestibility of the bread." "The whole art of milling has for its object the elimination of offal, and this has been strictly in accordance with scientific principles, and has resulted in the production of flour from which better bread can be made and also bread of a higher nutritive quality." In the circumstances it is fair to conclude that the case for "standard" bread has broken down, and that the experience of the navvies of Newcastle and the hard-working labourers, who prefer white bread to brown, is a safe guide to follow in selecting a bread for daily consumption. The usefulness of brown bread in certain conditions is unquestionable; the usefulness of "standard" bread is a dogmatic assertion not supported by facts.

Space forbids us to deal with the other interesting chapters in this valuable contribution to the dietetics of bread. We may merely mention that the authors have taken a catholic view of their subject and have passed in order before them all the various aspects of the baking and bread question, paying especial attention to the subject of adulteration—a matter which is of far greater importance than the fashion in "standard" bread. Their remarks are well worth consideration, and anyone who is interested in the subject should procure the book and study the matter at first hand. The volume is one which should have a place in every institutional and public library, and which should be perused by every member of the profession. Its technicalities render it caviare to the multitude, but we trust that the lay press, in reviewing it, will at least point out the great importance and value of the information it contains.

